# CAR‐NK cells from engineered pluripotent stem cells: Off‐the‐shelf therapeutics for all patients

**DOI:** 10.1002/sctm.21-0135

**Published:** 2021-11-01

**Authors:** Shi‐Jiang Lu, Qiang Feng

**Affiliations:** ^1^ HebeCell Corporation Natick Massachusetts USA

**Keywords:** CAR‐T, CAR‐NK, cellular immunotherapy, off‐the‐shelf therapeutics, pluripotent stem cells

## Abstract

Clinical success of adoptive cell therapy with chimeric antigen receptor (CAR) T cells for treating hematological malignancies has revolutionized the field of cellular immunotherapy. However, due to the nature of utilizing autologous T cells, affordability and availability are major hurdles, in addition to scientific challenges relating to CAR‐T therapy optimization. Natural killer (NK) cell is a specialized immune effector cell type that recognizes and kills targets without human leukocyte antigen (HLA) restriction and prior sensitization. CAR‐NK cells do not cause graft vs host disease and can be obtained from unrelated donors as well as pluripotent stem cells (PSC), representing an ideal off‐the‐shelf therapeutics readily available for patients. Furthermore, unlike cytotoxic T cells, NK cells specifically target and eliminate cancer stem cells, which are the cells causing relapse and metastasis. PSCs can be genetically manipulated and engineered with CARs at the pluripotent stage, which allows the establishment of permanent, stable, and clonal PSC‐CAR lines for the manufacture of unlimited homogenous CAR‐NK cells. Multiple master PSC‐CAR cell banks targeting a variety of antigens for cancer, viral infection, and autoimmune diseases provide inexhaustible cell sources for all patients. Development of a next‐generation 3D bioreactor platform for PSC expansion and NK cell production overcomes major barriers related to cost and scalability for CAR‐NK product.

## Graphical abstract

The establishment of a master pluripotent stem cell (PSC)‐CAR cell bank targeting a variety of specific antigens will provide inexhaustible cell sources for the manufacture of truly off‐the‐shelf CAR‐NK cells for all patients of cancer, infectious, and autoimmune diseases.

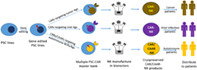

**Significance statement**

Critical to the success of chimeric antigen receptor (CAR) engineered immune effector cell therapies will be the industrialization—converting the technologies into universal and cost‐effective therapies for a large number of patients. Autologous CAR‐T cell therapy has been successful for the treatment of certain blood malignancies; limited cell availability and high manufacturing cost make it difficult to be used for most patients. The development of a 3D PSC‐CAR‐NK platform will allow scalable, reproducible, and efficient production of homogenous functional CAR‐NK cells, which can be rapidly deployed worldwide for all patients.

## 1 CAR‐T THERAPY: PROMISE AND CHALLENGES

### 1.1 The promise

Adoptive cell transfer (ACT) for the treatment of human diseases has advanced rapidly in the past few years, especially for cancer. Chimeric antigen receptor (CAR), artificially engineered to express on the surface of immune effector cells, functions as a GPS and redirects effector cells toward their targets such as tumors^1^ and has revolutionized the ACT therapeutic field. The success of autologous CD19 CAR‐T cell therapy against hematological malignancies, such as chronic lymphocytic leukemia (CLL), acute lymphocytic leukemia (ALL), and non‐Hodgkin lymphoma (NHL), represents one of the most exceptional breakthroughs in cancer immunotherapy in the past decade.^2^, ^3^, ^4^, ^5^ This success and the approval of CD19 CAR‐T cell therapy by the U.S. Food and Drug Administration (FDA) have prompted the exploration, all over the world, of CAR‐T clinical trials targeting different antigens for different tumors.^6^

### 1.2 The challenges

The success of CD19 CAR‐T therapy, however, is not accomplished without side effects. The eradication of tumors by CD19 CAR‐T cells leads to the release of several inflammatory cytokines, including IL‐6 and TNF‐α, which is termed cytokine release syndrome (CRS). In most patients, CRS is associated with fever and hemodynamic compromise, which can be fatal without proper intervention. Neurotoxicity has also been observed concurrent with or following CRS in some patients treated with CD19 CAR T cells, the cause of which is not clear although there are reports that CD19 CAR‐T cells may penetrate the blood‐brain barrier and attack cells expressing CD19.^2^, ^3^, ^4^, ^5^, ^7^

The translation of CAR‐T therapy beyond B‐cell malignancies to other tumors (including other blood malignancies) still faces several obstacles that need to be addressed^6^, ^7^, ^8^, ^9^:

*First, specificity*: most CARs are targeting tumor‐associated antigens (TAAs) that are also expressed on normal tissues, which leads to severe toxicity. Although there are different approaches to minimize the scale of toxicity, on‐target/off‐tumor toxicity can be solved completely by identifying neoantigens, which are derived from tumor‐specific gene mutations (drivers), as their formation and expression are restricted to malignant tumor cells.^7^, ^10^
*Second, resistance/escape*: as TAAs are not required for tumor cell survival, loss of TAA expression is the major cause of development of resistance to CAR‐T therapies. A strategy to circumvent tumor resistance/escape is to target multiple TAAs simultaneously by constructing bivalent‐ or multivalent‐specific CAR‐T cells, such that only tumor cells that lack expression of all target molecules would escape CAR‐T therapy.^11^ Other approaches are also explored with some success.^7^, ^12^, ^13^
*Third, solid tumors*: although some satisfactory efficacy results were observed in some leukemic patients, especially with CD19 CAR‐T therapy for B‐cell lymphoma and BCMA CAR‐T for myeloid myeloma, comparable efficacy in solid tumors has not yet been achieved. These disappointing results can be attributed to several factors, including^6^, ^7^, ^8^, ^9^ the inability of the CAR T cell to infiltrate into solid tumors, immunosuppressive molecules, and cells in the tumor microenvironment (TME), heterogenous expression of target antigens in tumor cells, and intrinsic T cell dysfunction and exhaustion. Regional or intratumor injection instead of systemic administration has been shown to be superior at least in animal model studies.^14^ Combination of CAR‐T with modulators of the TME, such as checkpoint inhibitors, have shown efficacy in some cancers. Other strategies include (a) depleting Tregs and myeloid‐derived suppressor cells with blocking antibodies, (b) engineering CAR T cells to secrete extracellular matrix‐degrading enzymes that degrade cancer‐associated fibroblasts, (c) altering CAR‐T cell metabolic profiles to enhance their function in hostile TME, and (d) constructing bivalent/multivalent CAR‐T cells. Some of these approaches are now undergoing clinical evaluation.^7^, ^15^, ^16^
*Fourth, manufacturing*: all the above CAR T optimizations are biological challenges, and the manufacturing of CAR‐T cells is another challenge. Most CAR‐T cells are derived from patients' own peripheral blood mononuclear cells (PBMCs), which requires a bespoke manufacturing process for every patient after leukapheresis. The cost of the process, as well as potential manufacturing failure and delay (~3 weeks), renders the affordability and availability less favorable. Furthermore, individual to individual variations make the CAR‐T therapeutics inconsistent and could result in reduced efficacy in some patients owing to their T cell dysfunction.^7^, ^8^ By simultaneous introduction of CAR and disruption of T‐cell receptor (TCR) and CD52 in T cells, Qasim et al reported the generation of allogenic CAR‐T cells, which are able to evade host immunity and avoid graft vs host disease (GvHD) for the treatment of unmatched patients.^17^ The allogenic off‐the‐shelf CAR T cells from healthy donors is an alternative strategy to address the complexities of manufacturing and high costs of individualized CAR‐T cell products, but there is still the risk of GvHD caused by less than even 1% of TCR+ cells in TCR knock‐out CAR‐T cell preparation, and early‐phase clinical trials have met some hurdles.^18^, ^19^ On July 6, 2020, the FDA issued a clinical hold for the phase I trial of a universal CAR‐T product, UCARTCS1A (ClinicalTrials.gov Identifier: NCT04142619) developed by Cellectis, due to the death of one participant caused by cardiac arrest.^20^ To realize the therapeutical potential of engineered immune effector cells, other types of cells, including dendritic cells, macrophages, red blood cells, mesenchymal stem cells, and endothelial progenitor cells, have been explored^21^; however, one type of immune cells—the natural killer (NK) cell—has been considered the most promising of cells for immunotherapy.

## 2 NK CELLS AS AN IDEAL ACT PRODUCT

### 2.1 NK cells as allogenic therapeutics

NK cells are a type of cytotoxic lymphocyte of the innate immune system and are the gatekeeper of the immune system, with the ability to recognize and kill targets without HLA restriction or prior sensitization, allowing for a much faster immune reaction.^22^ NK cell cytotoxicity is regulated by the balance of activating and inhibitory receptors, which can efficiently kill abnormal cells in case activating ligands are present and signals for inhibitory receptors are absent. Different from T cells, NK cells are capable of killing cells that are missing “self” markers of MHC I antigens, whereas harmful cells missing MHC I markers cannot be detected and destroyed by T cells.^22^ Most importantly, adoptive NK cell transfer does not require strict HLA matching and lacks the potential to cause GvHD, a critical risk imposed by any T cell immunotherapy.^23^ Therefore, NK cells can be off‐the‐shelf allogeneic therapeutics.

### 2.2 CAR‐NK vs CAR‐T

Similar to T cells, NK cells have been engineered with CAR expressed on the surface to target a specific antigen, enhancing their natural cytotoxicity with specificity.^24^, ^25^ Recently, Liu et al^26^ reported the interim results of a phase I/II trial using umbilical cord blood (UCB)‐derived, CD19‐targeted CAR‐NK therapy in patients with relapsed or refractory CD19+ cancers. Eight of 11 patients had an objective response to treatment without development of major toxic effect, demonstrating the safety and efficacy of CAR‐NK against hematopoietic malignancies.^26^ Compared with CAR‐T cells, CAR‐NK cells have the following advantages^27^ (Table 1): (a) Unlike CAR‐T cells, CAR‐NK cells retain an intrinsic capacity to recognize and target abnormal cells through their native receptors, making the escape of abnormal cells through downregulation of CAR targeted antigens less likely; (b) CAR‐NK cells do not undergo clonal expansion and cause CRS, neurotoxicity and other side effects that have been observed in many CAR‐T clinical trials; (c) CAR‐NK cells do not need to be patient matched, therefore cells from healthy donor PBMC or UCB can be used for the manufacture of allogenic CAR‐NK cells, which render it more affordable and feasible; (d) By default, NK cells have been shown to preferentially target and kill cancer stem cells as they are quiescent and express low levels of MHC I.^28^, ^29^ This is advantageous because cancer stem cells are resistant to both traditional chemotherapy and radiotherapy, as well as immunotherapy, which leads to relapse and metastasis.

**TABLE 1:** Comparison of CAR‐T vs donor‐CAR‐NK and PSC‐CAR‐NK cell products

	CAR‐T	Donor CAR‐NK	PSC‐CAR‐NK
Cell source	Autologous peripheral blood	Allogenic PB and cord blood	Allogenic iPSC and hESC cells, unlimited
Manufacture time, cost, and deliverable doses	Time consuming, high cost, single dose	Off‐the‐shelf, low cost, limited multiple doses	Off‐the‐shelf, very low cost, unlimited doses for all patients
Product identity	Heterogenous	Heterogenous	Homogenous and consistent
Gene editing/CAR	Random and variable	Random and variable, massive cells death with viral infection	Uniform. CAR/gene editing in PSCs—100% CAR/gene‐edited NK cells
Cancer stem cell	No preferential targeting	Yes, preferential targeting	Yes, preferential targeting
Killing mechanism	CAR‐mediated cell killing	Both CAR and NK receptor‐mediated cell killing	Both CAR and NK receptor‐mediated cell killing, plus gene editing
Safety: CRS	High	Low	Low
GvHD	High, with allogenic cells	Low, even with allogenic cells	Low, even with allogenic cells
Master cell bank	No	No	Yes: PSC‐CAR cell banks for variety of targets

### 2.3 Donor‐based CAR‐NK: Challenges

The main sources of donor NK cells are peripheral blood (PB) and UCB, and their concentration of 1 × 10^8^ cells/L comprises ∼1‐2% of total WBCs, or 0.01‐0.02% of all cells in the PB.^30^ Although in vitro exponential expansion *with/without* artificial antigen presenting cells (aAPC) from a single donor will be able to provide enough cells for a single or limited number of patients, the doses are still limited from a single‐donor phlebotomy,^31^, ^32^, ^33^ which is far less for an ideal off‐the‐shelf allogeneic commercial therapeutic product. Functional and expansion variations among different donors add another burden for batch to batch validation as well as manufacture delay, which lead to inconsistent clinical outcome. Furthermore, transfection of NK cells with viral CAR‐constructs was associated with low or variable levels of transgene expression and unfavorable effects on cell viability, limiting other genetic engineering to improve the efficacy of CAR‐NK cells.^24^ NK cell lines, such as NK9‐2, have been pursued for clinical applications. These cells are cancerous in nature and need to be irradiated before infusion into patients, compromising their cancer killing capabilities.^34^

As an ideal cellular immunotherapy product, cells should possess high specificity on targets with minimized off‐target toxicities; be prepared from sustainable sources so every patient receives the same cell product; be able to be stored and ready for patient administration whenever needed, not be prepared for each individual patient; and be centrally manufactured in a current good manufacturing practice (cGMP) facility and be able to distribute worldwide. CAR‐NK cells from donor sources fulfill most of above criteria but miss the most important property for an allogenic off‐the‐shelf cell product—sustainability. One potential source of CAR‐NK cells is pluripotent stem cells (PSC) including human embryonic stem cells (hESC) and induced pluripotent stem cells (iPSC), which may circumvent the challenges associated with donor‐derived CAR‐NK cells.

## 3 PSCs AS A SOURCE OF ALLOGENIC OFF‐THE‐SHELF CAR‐NK CELLS

### 3.1 In vitro differentiation of PSCs toward NK cells

Both hESCs and iPSCs can be propagated and expanded in vitro indefinitely, providing a potentially inexhaustible and donorless source of hematopoietic cells for human therapy. Differentiation of PSCs into hematopoietic cells has been extensively investigated in vitro for the past two decades. Hematopoietic precursors as well as mature, functional progenies representing erythroid, myeloid, macrophage, megakaryocytic and lymphoid lineages have been identified in the differentiating PSC cultures.^35^, ^36^, ^37^, ^38^, ^39^, ^40^ The differentiation of PSCs into lymphoid lineage cells has proven to be more difficult than their differentiation into other lineages. Dan Kaufman's group has pioneered the differentiation of NK cells from both hESCs and iPSCs. The 2‐step differentiation procedure starts with PSCs on feeder cells to generate CD34+ hematopoietic progenitor cells,^38^ which was later optimized to culture PSCs directly on matrix coated petri dishes as adherent monolayer culture.^39^ CD34+ cells (5‐15% of total cells depend on culture conditions) were then isolated from the culture and transferred onto engineered stroma in medium containing mixture of cytokines for approximately 2 weeks, which generated a highly enriched homogenous population of CD45+ CD56+ CD94+ NK cells.^38^, ^39^ Both in vitro and in vivo studies showed that PSC‐NK cells were capable of secreting IFNγ in response to cytokine stimulation and harbored potent natural cytotoxicity against multiple types of cancer cells as well as displayed antitumor activity in xenograft mouse models.^38^, ^39^, ^41^, ^42^ Several groups have also successfully differentiated PSCs into NK cells that are similar in phenotype such as NK‐associated receptor phenotype and effector function when compared with PB‐NK cells.^43^, ^44^

### 3.2 CAR‐NK cells derived from PSCs: Advantages

PSCs provide a completely novel pathway for the generation of gene engineered cell products such as NK cells. There are several advantages of PSC‐CAR‐NK compared with donor based CAR‐NK cells (Table 1). First, CAR construct can be inserted into undifferentiated PSCs to establish permanent, stable, and clonal PSC‐CAR lines, which can be used for unlimited manufacture of homogenous CAR‐NK cells, a true off‐the‐shelf and consistent product for all patients.^41^ As CAR is engineered at the pluripotent stage of PSCs, there is no need to transfect NK cells with viral vectors, avoiding mass cell death caused by viral infection and uncontrollable variation of gene insertion and expression. Li et al reported iPSC‐CAR‐NK cells with improved killing of ovarian cancer cells in vitro and in vivo.^41^ Based on the above technology, several iPSC‐CAR‐NK cells are currently in clinical trials to evaluate their safety and efficacy for both hematological and solid tumors by Fate Therapeutics. Second, CARs targeting different antigens can be introduced into PSCs to establish PSC‐CAR banks, covering targets for a variety of cancers, viral infection, and autoimmune diseases. The master PSC‐CAR cell bank will provide inexhaustible cell sources for the manufacturing of truly off‐the‐shelf CAR‐NK cells for all patients. Third, it allows precise genetic engineering at PSC stage to generate fully genetic edited clonal lines, shaping the phenotype and functionality of the resulting products and overcoming the various limitations associated with NK therapies. These manipulations include (a) introduction of mutated versions of high affinity CD16 receptor conferring resistance to enzymatic cleavage with improved antibody‐dependent cell‐mediated cytotoxicity (ADCC) and cancer cell killing^45^ which is undergoing phase I clinical trials in adults with hematologic malignancies by Fate Therapeutics; (b) incorporation of fused cytokine receptor‐ligand receptors such as IL15‐IL15r fusion receptor (RLI) to improve in vivo persistence and expansion^46^; and (c) knock‐out of checkpoint inhibitors such as CISH gene to improve metabolic fitness and cytotoxicity.^47^ These advantages offer new solutions for obstacles associated with conventional NK therapies.^46^

### 3.3 Manufacturing challenge of current PSC‐NK cells

The manufacture of safe and effective PSC‐NK cells will offer a viable alternative and future replacement of donor‐NK sources. As summarized here, significant progress has been made toward this end by genetic manipulating PSCs and driving them toward NK lineage development. Despite many exciting advances with in vitro culture systems, the requirement for initial monolayer or forced embryoid body (EB) formation in specialized 96‐well plate and the need to isolate rare CD34/CD45 double positive cells as well as two (at least one in some instance) different types of stroma coculture^38^, ^39^, ^41^, ^42^, ^43^ limits the utility of this approach for industrial‐scale, cost‐effective and GMP grade production of PSC‐generated NK cells.

### 3.4 3D platform for industrial scale production of PSC‐NK cells

**FIGURE 1:**
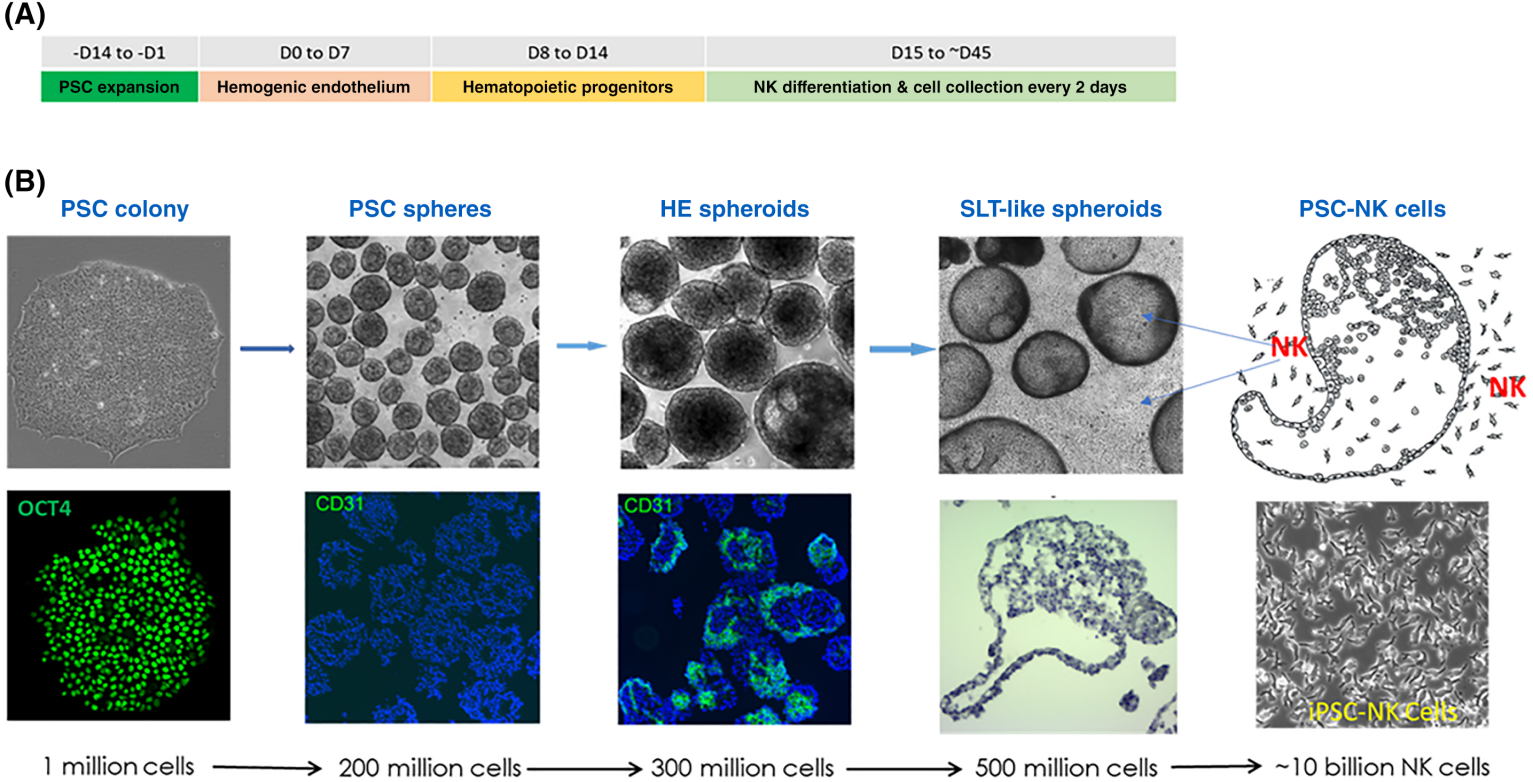
Sequential differentiation of pluripotent stem cells (PSC) toward hemogenic endothelial (HE), hematopoietic progenitors and NK cells in 3D bioreactor. A, Scheme shows approximately times and stepwise strategy used for 3D‐PSC differentiation into hemogenic endothelium, hematopoietic progenitors, and NK cells. B, Morphology and specific gene expression at different stages. SLT, secondary lymphoid tissues

**FIGURE 2:**
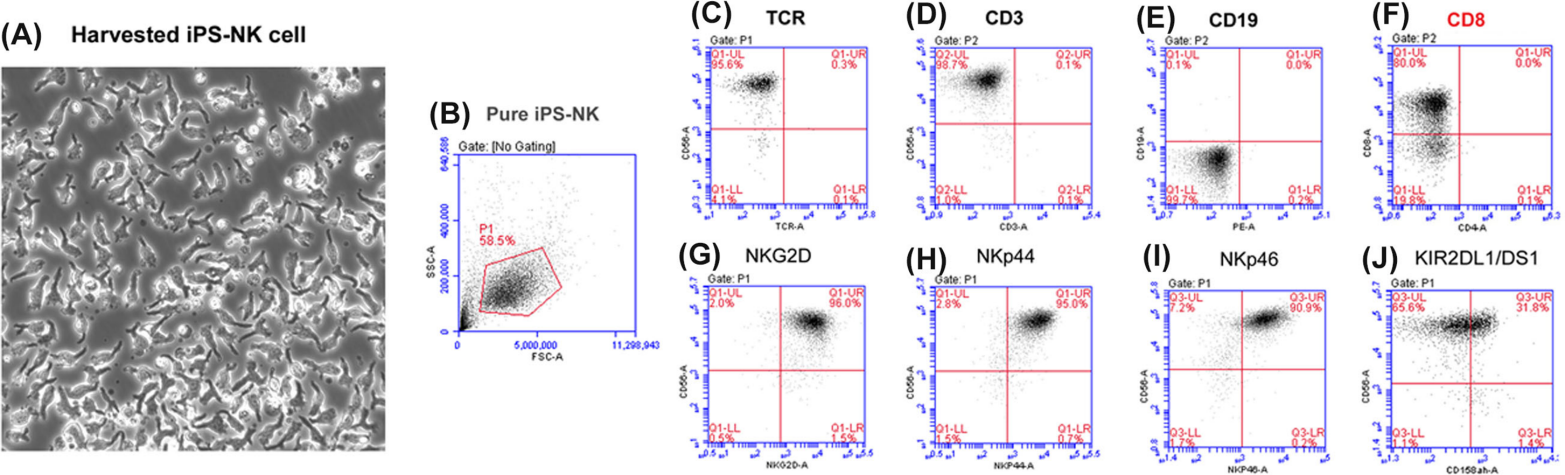
Flow cytometry analysis of iPS‐NK cells. A, morphology of iPS‐NK cells collected from 3D‐bioreactors without purification. B, Representative forward and site scattering plots of iPS‐NK cells. C‐J, Representative flow cytometry plots of iPS‐NK cells for (C) CD56 (y) and TCR(x); (D) CD56(y) and CD3(x); (E) CD19 (y) and PE conjugated antibody control (x); (F) CD8(y) and CD4(x); (G) CD56(y) and NKG2D (x); (H) CD56(y) and NKp44(x); (I) CD56(y) and NKp46(x); and (J) CD56(y) and KIR2DL1/DS1(x)

We have developed a novel 3D‐bioreactor platform to efficiently convert human PSCs into highly pure NK cells continuously in a single bioreactor (Figure 1).^48^ This differentiation system uses a defined serum‐free medium and eliminates the use of any feeder cells. First, human PSCs were adapted from 2D monolayer culture to 3D spheres and expanded in a bioreactor, then 3D PSC‐spheres were induced toward mesoderm lineage differentiation, which convert 60‐70% PSCs into hemogenic endothelial progenitors (CD31+ CD144+ CD34+) in a week. Hemogenic endothelial cells were then switched to hematopoietic and NK cell differentiation conditions for up to 35‐45 days. Starting from approximately day 15, NK cells were released from these 3D‐spheres and lasted for ~4 weeks, and the released NK cells were harvested by simple centrifugation (Figure 1). The released PSC‐NK cells display a distinctively homogenous morphology and express gene signatures characteristic to typical NK cells.^48^ Over 95% of collected cells express CD56 and activating receptors of NKG2D, NKp44 and NKp46 (Figure 2). These CD56+ PSC‐NK cells do not express T cell markers of TCR and CD3, nor B cell marker CD19; whereas more than 60% CD56+ PSC‐NK cells also express CD8α (Figure 2) as compared with ≈30% of PB CD56+ NK cells, the increase of which is associated with disease regression in AIDS patients.^49^ We have reproducibly generated ~10^10^ pure NK cells with a 300 mL bioreactor and this process has been repeated with multiple hESC and iPSC lines with/without gene editing or CAR insertion.

In vitro assays showed that PSC‐NK cells produced by 3D‐bioreactors were capable of secreting IFNγ, expressing CD107a (degranulation) in response to stimulation, and also displayed potent natural cytotoxicity against multiple tumor cells including leukemia cells as well as pancreatic, ovarian, colorectal and lung cancer cells. We also demonstrated that PSC‐NK cells harbored anti‐virus activity, and specifically killed normal cells infected with influenza A, Dengue, Zika, and HIV viruses, but not uninfected normal cells.^48^

This 3D PSC‐NK manufacture platform uses defined materials without serum or feeder cells, and is scalable and reproducible for generation of homogenous functional NK cells. With the establishment of permanent master PSC‐CAR cell lines, this novel technology platform will provide inexhaustible cell sources for the generation of off‐the‐shelf CAR‐NK cells suitable for treatment of a large numbers of patients, which can revolutionize the field of cellular immunotherapy.

## 4 THE FUTURE PERSPECTIVE

Human PSCs promise to provide a potentially inexhaustible source of CAR‐NK cells for immunotherapy. However, before PSC‐CAR‐NK cells can be used in the clinic, it is important to understand the risks and challenges associated with these therapeutic products.

One of the most important characteristics of undifferentiated PSCs is teratoma formation.^50^ This characteristic is a serious concern, because if undifferentiated or under‐differentiated PSCs remain in the final CAR‐NK product, tumor formation may occur. Fortunately, several groups have demonstrated that undifferentiated human iPSCs are the target of NK cells both in vitro and in vivo.^51^, ^52^, ^53^, ^54^ Specifically, injection of human iPSCs in immune deficient mice failed to form teratoma when these animals were reconstituted with allogenic or autologous PBMC or purified NK cells alone; whereas teratomas were observed in animals reconstituted with autologous PBMCs depleted off NK cells,^53^ confirming NK cells prevent the formation of teratoma. We also observed that coculture of human iPSCs with autologous iPSC‐NK cells overnight caused mass iPSC death, demonstrating pluripotent iPSCs, if present, cannot survive the hostile surroundings of a highly concentrated iPSC‐NK cell population. Furthermore, various suicide genes have been transduced into PSCs that can eliminate undifferentiated PSCs and prevent teratoma formation in PSC‐derived cell therapy products.^55^, ^56^ The approval of multiple iPSC‐NK products (Fate Therapeutics) by the FDA clearly paves the way for clinical application of PSC‐CAR‐NK products.

Due to the ethical concerns, very limited new hESC lines have been derived and most existing hESC lines have not been derived under GMP conditions by utilizing undefined animal products and feeder cells, which could potentially contaminate them with animal pathogens and viruses. Although iPSC lines can be reprogrammed from many types of somatic cells, only a limited number of GMP compliant iPSC lines are currently available mainly due to the regulatory requirements for donors and absence of qualified materials and reagents for the generation of iPSCs. Therefore, in order to comply with government regulations and fulfill the promise of PSC‐CAR‐NK therapeutics, new hESC and iPSC lines will need to be produced under GMP conditions using defined materials that can be traced back to the origin or using xeno‐free products in manufacturing PSC‐CAR‐NK cell products for clinical application.

**FIGURE 3:**
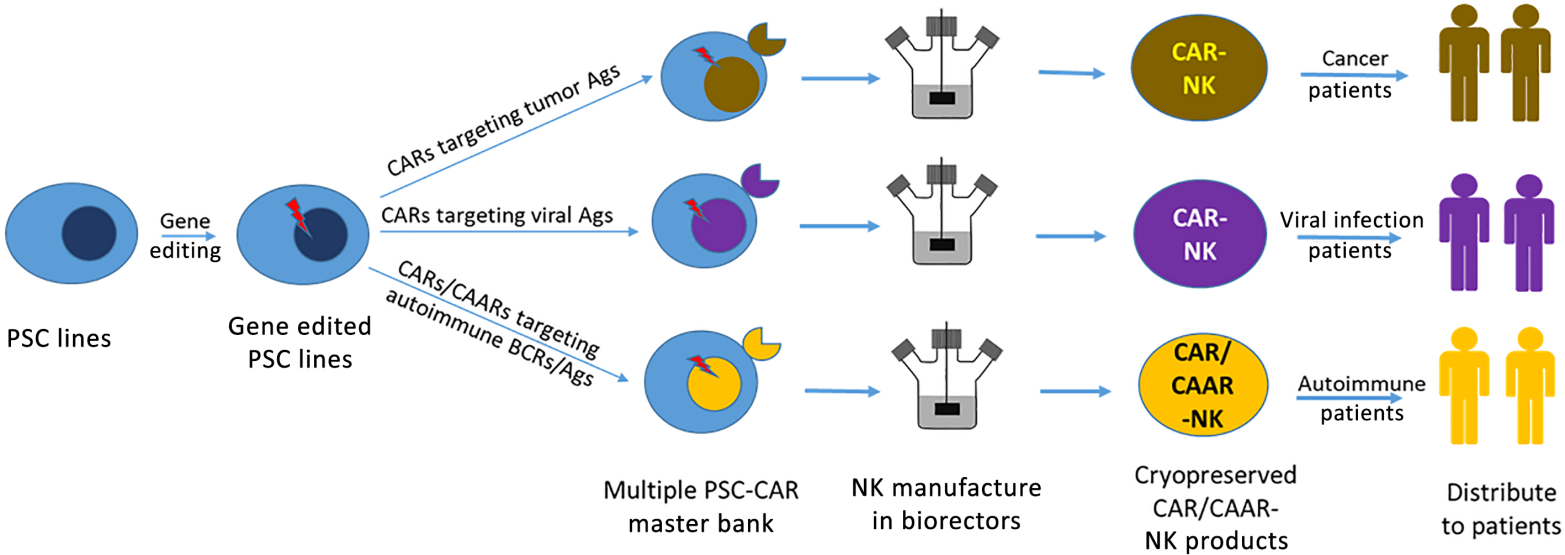
Genetically engineered PSC‐CAR‐NK cell therapeutic strategies for cancer, viral infection, and autoimmune diseases

Successful clinical application of cell products derived from PSCs will require efficient and controlled differentiation of PSCs toward a specific cell type with the generation of a homogeneous cell population. Our 3D PSC‐NK technology provides a scalable, consistent, efficient, and cost‐effective manufacturing platform for the generation of homogenous functional NK cells. As illustrated in Figure 3, GMP grade PSC lines can be genetically manipulated at the pluripotent stage to enhance the persistence and cytotoxicity of final NK cell products. Gene edited PSC lines can be further engineered with CARs targeting different cancer antigens and viral antigens^57^, ^58^ or autoreactive T cells as well as chimeric autoantibody receptor (CAAR) against autoreactive B cells.^34^, ^59^, ^60^ The establishment of multiple master PSC‐CAR cell banks targeting different specific antigens will provide inexhaustible cell sources for the manufacture of truly allogenic off‐the‐shelf CAR‐NK cells, which can be rapidly deployed worldwide for patients of cancer, infection and autoimmune diseases. Future work aims toward developing larger (>5 L) bioreactors and logistics, with ultimate goal of making CAR‐NK products affordable and available for ordinary patients and as easy to handle as conventional drugs.

## CONFLICT OF INTEREST

S.J.L. is the co‐founder and chief executive officer and Q.F. is the co‐founder and chief scientific officer of HebeCell Corporation. Both S.J.L. and Q.F. declare employment and stock ownership at HebeCell Corporation.

## AUTHOR CONTRIBUTIONS

S.J.L. and Q.F.: conception/design and manuscript writing.

## Data Availability

Data sharing is not applicable to this article as no new data were created or analyzed in this study.
